# (*Z*)-3-Anilino-1,3-diphenylprop-2-en-1-one

**DOI:** 10.1107/S1600536808016711

**Published:** 2008-06-21

**Authors:** Li-Ping Zhang, Lin-Juan Wei, Ming-Qing Chen, Zhan-Hui Zhang

**Affiliations:** a School of Chemical and Materials Engineering, Jiangnan University, 1800 Lihu Road, Wuxi 214122, Jiangsu, People’s Republic of China; b School of Chemistry and Materials Science, Hebei Normal University, 113 Yuhua Road, Shijiazhuang 050000, Hebei, People’s Republic of China

## Abstract

In the title compound, C_21_H_17_NO, the phenyl ring directly linked to the carbonyl group is oriented at an angle of 7.3 (2)° with respect to the aniline ring, and at an angle of 55.6 (2)° with respect to the other phenyl ring. There is an intra­molecular hydrogen bond involving the NH group and the carbonyl O atom. The crystal structure is stabilized by weak C—H⋯π inter­actions, which link the mol­ecules into a herringbone arrangement.

## Related literature

For related literature see: Dondoni & Perrone (1993 ▶); Ferraz *et al.* (1995 ▶); Michael *et al.* (2001 ▶); Azzaro *et al.* (1981 ▶); Alberola *et al.* (1999 ▶); Chaaban *et al.* (1979 ▶); Augusti & Kascheres (1993 ▶); Bejan *et al.* (1998 ▶); Eberlin & Kascheres (1988 ▶); Greenhill (1977 ▶); Michael *et al.* (1999 ▶); Elassar & El-Khair (2003 ▶); Zhang *et al.* (2006 ▶).
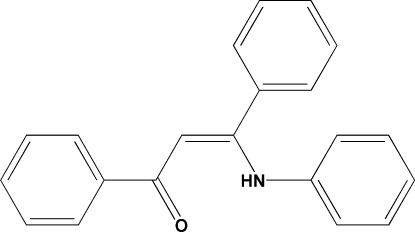


## Experimental

### 

#### Crystal data

C_21_H_17_NO
*M* *_r_* = 299.36
Monoclinic, 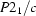

*a* = 15.880 (7) Å
*b* = 6.034 (3) Å
*c* = 18.401 (8) Å
β = 114.433 (7)°
*V* = 1605.4 (13) Å^3^
*Z* = 4
Mo *K*α radiation
μ = 0.08 mm^−1^
*T* = 294 (2) K
0.24 × 0.20 × 0.14 mm

#### Data collection

Bruker SMART CCD area-detector diffractometer
Absorption correction: multi-scan (*SADABS*; Bruker, 2000 ▶) *T*_min_ = 0.96, *T*_max_ = 0.98
7511 measured reflections
2774 independent reflections
1580 reflections with *I* > 2σ(*I*)
*R*_int_ = 0.051

#### Refinement

*R*[*F* ^2^ > 2σ(*F* ^2^)] = 0.079
*wR*(*F* ^2^) = 0.249
*S* = 1.04
2774 reflections
209 parameters
H-atom parameters constrained
Δρ_max_ = 0.24 e Å^−3^
Δρ_min_ = −0.23 e Å^−3^

### 

Data collection: *SMART* (Bruker, 2000 ▶); cell refinement: *SAINT* (Bruker, 2000 ▶); data reduction: *SAINT*; program(s) used to solve structure: *SHELXS97* (Sheldrick, 2008 ▶); program(s) used to refine structure: *SHELXL97* (Sheldrick, 2008 ▶); molecular graphics: *SHELXTL* (Sheldrick, 2008 ▶); software used to prepare material for publication: *SHELXTL*.

## Supplementary Material

Crystal structure: contains datablocks global, I. DOI: 10.1107/S1600536808016711/su2060sup1.cif

Structure factors: contains datablocks I. DOI: 10.1107/S1600536808016711/su2060Isup2.hkl

Additional supplementary materials:  crystallographic information; 3D view; checkCIF report

## Figures and Tables

**Table 1:** Hydrogen-bond geometry (Å, °)

*D*—H⋯*A*	*D*—H	H⋯*A*	*D*⋯*A*	*D*—H⋯*A*
N1—H1⋯O1	0.86	1.94	2.643 (5)	138
C18—H18⋯*Cg*^i^	0.93	2.84	3.627 (1)	142

Symmetry code: (i) 
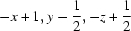
. *Cg* is the centroid of the C16–C21 ring.

**Fig. 1.**
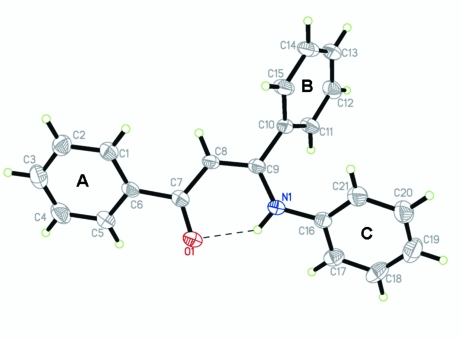
The molecular structure of compound (I), showing the atomic numbering scheme and the displacement ellipsoids drawn at the 30% probability level. The intramolecular N-H···O hydrogen bond is shown as a dashed line.

**Fig. 2.**
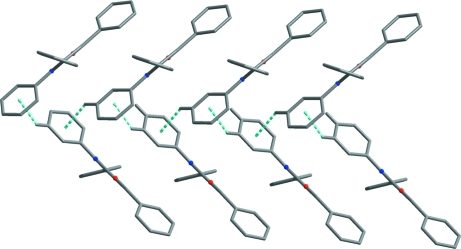
The molecular packing of compound (I), showing the C—H···π interactions (dashed lines).

## supplementary crystallographic information

### Comment

β-enamino ketones are a highly versatile class of intermediates for the synthesis of natural therapeutic and biologically active analogues (Dondoni & Perrone, 1993; Ferraz *et al.*, 1995; Michael *et al.*, 2001; Azzaro *et al.*, 1981). Pyrroles, oxazoles, pyridinones, quinolines, dibenzodiazepines have also been prepared from enaminones (Alberola *et al.*, 1999; Chaaban *et al.*, 1979; Augusti & Kascheres, 1993; Bejan *et al.*, 1998; Eberlin & Kascheres, 1988). It is therefore not surprising that many synthetic methods have been developed for the preparation of these compounds (Greenhill, 1977; Michael *et al.*, 1999; Elassar  & El-Khair, 2003). During our development of new environmentally friendly methodologies for the preparation of β-enamino ketones (Zhang *et al.*, 2006), we synthesized the title compound, (I), the structure of which is reported here.

The molecular structure of compound (I) is illustrated in Fig. 1. The geometry of the enamine double bond is *Z*, with hydrogen bonding of the enamine N—H to the carbonyl oxygen atom (Table 1). Phenyl ring A (C1-C6) forms dihedral angles of 7.3 (2)° and 55.6 (2)° with the aniline ring C (C16-C21) and the phenyl ring B (C10-C15), respectively. As in other β-enamino ketones compound (I) displays electron delocalization, as shown by the comparison of the N1—C9 [1.347 (6)°] and N1—C16 [1.417 (6) Å] bond lengths.

The crystal structure of compound (I) is stabilized by weak C—H···π interactions, which link the molecules into a herringbone chain (Fig. 2). The distance of the H atom to the centroid of the benzene ring is 2.834 (10) Å.

### Experimental

A mixture of the 1,3-diphenylpropane-1,3-dione (5 mmol), aniline (5 mmol) and InBr_3_ (0.05 mmol) was stirred at room temperature for 6 h. After completion of the reaction, the reaction mixture was diluted with H_2_O (10 ml) and extracted with EtOAc(210 ml). The combined organic layers were dried, concentrated, and then purified by column chromatography on SiO_2_ with ethyl acetate-cyclohexane (1: 8), giving a yellow-orange solid (yield 68%). Mp 96–97°C; IR (neat, cm^-1^): ν 3053, 1592, 1568, 1475, 1441, 1323, 1212, 1079, 1053, 1022, 904, 841, 697 ^1^H NMR (CDCl_3_, 300 MHz): δ 6.09 (s, 1H), 6.78 (d, 2H), 6.96–7.00 (m, 1H), 7.09–7.14 (m, 2H), 7.26–7.50 (m, 8H), 7.96 (d, 2H), 12.90 (br s, 1H, NH). ^13^C NMR (CDCl_3_, 75 MHz): δ 97.0, 123.1, 124.2, 127.2, 128.2, 128.5, 128.7, 129.6, 131.3, 135.8, 139.4, 139.8, 161.4, 189.6. ESI-MS: 300 (*M*+1)^+^ Elemental Anal. Calcd. for C_21_H_17_NO: C, 84.25; H, 5.72; N, 4.68. Found: C, 84.48; H, 5.82; N, 4.45. Single crystals of (I), suitable for X-ray diffraction analysis, were obtained from ethyl acetate-cyclohexane by slow evaporation at room temperature.

### Refinement

H atoms were placed in geometrically idealized positions and constrained to ride on their parent atoms, with N—H = 0.86 Å, C—H = 0.93 - 0.97 Å, and *U*_iso_(H) = 1.5*U*_eq_(CH_3_) or 1.2*U*_eq_(C, *N*).

### Crystal data

**Table:**

C_21_H_17_NO	*F*_000_ = 632
*M**_r_* = 299.36	*D*_x_ = 1.239 Mg m^−^^3^
Monoclinic, *P*2_1_/*c*	Mo *K*α radiation λ = 0.71073 Å
Hall symbol: -P 2ybc	Cell parameters from 1702 reflections
*a* = 15.880 (7) Å	θ = 2.3–23.8º
*b* = 6.034 (3) Å	µ = 0.08 mm^−^^1^
*c* = 18.401 (8) Å	*T* = 294 (2) K
β = 114.433 (7)º	Block, yellow
*V* = 1605.4 (13) Å^3^	0.24 × 0.20 × 0.14 mm
*Z* = 4	

### Data collection

**Table:**

Bruker SMART CCD area-detector diffractometer	2774 independent reflections
Radiation source: fine-focus sealed tube	1580 reflections with *I* > 2σ(*I*)
Monochromator: graphite	*R*_int_ = 0.052
*T* = 294(2) K	θ_max_ = 25.0º
phi and ω scans	θ_min_ = 2.3º
Absorption correction: multi-scan(SADABS; Bruker, 2000)	*h* = −17→18
*T*_min_ = 0.96, *T*_max_ = 0.98	*k* = −7→7
7511 measured reflections	*l* = −17→21

### Refinement

**Table:**

Refinement on *F*^2^	Hydrogen site location: inferred from neighbouring sites
Least-squares matrix: full	H-atom parameters constrained
*R*[*F*^2^ > 2σ(*F*^2^)] = 0.079	*w* = 1/[σ^2^(*F*_o_^2^) + (0.1119*P*)^2^ + 1.614*P*] where *P* = (*F*_o_^2^ + 2*F*_c_^2^)/3
*wR*(*F*^2^) = 0.249	(Δ/σ)_max_ < 0.001
*S* = 1.05	Δρ_max_ = 0.24 e Å^−^^3^
2774 reflections	Δρ_min_ = −0.23 e Å^−^^3^
209 parameters	Extinction correction: SHELXL97, Fc^*^=kFc[1+0.001xFc^2^λ^3^/sin(2θ)]^-1/4^
Primary atom site location: structure-invariant direct methods	Extinction coefficient: 0.021 (4)
Secondary atom site location: difference Fourier map	

### Special details

**Table:**

Geometry. All e.s.d.'s (except the e.s.d. in the dihedral angle between two l.s. planes) are estimated using the full covariance matrix. The cell e.s.d.'s are taken into account individually in the estimation of e.s.d.'s in distances, angles and torsion angles; correlations between e.s.d.'s in cell parameters are only used when they are defined by crystal symmetry. An approximate (isotropic) treatment of cell e.s.d.'s is used for estimating e.s.d.'s involving l.s. planes.
Refinement. Refinement of *F*^2^ against ALL reflections. The weighted *R*-factor *wR* and goodness of fit *S* are based on *F*^2^, conventional *R*-factors *R* are based on *F*, with *F* set to zero for negative *F*^2^. The threshold expression of *F*^2^ > σ(*F*^2^) is used only for calculating *R*-factors(gt) *etc*. and is not relevant to the choice of reflections for refinement. *R*-factors based on *F*^2^ are statistically about twice as large as those based on *F*, and *R*- factors based on ALL data will be even larger.

### Fractional atomic coordinates and isotropic or equivalent isotropic displacement parameters (Å^2^)

**Table:**

	*x*	*y*	*z*	*U*_iso_*/*U*_eq_	
O1	0.2315 (2)	0.0638 (5)	0.19524 (16)	0.0578 (9)	
N1	0.3124 (2)	−0.1522 (6)	0.11657 (19)	0.0490 (9)	
H1	0.3021	−0.1332	0.1585	0.059*	
C1	0.1066 (3)	0.5369 (7)	0.0895 (2)	0.0511 (11)	
H1A	0.1389	0.5646	0.0582	0.061*	
C2	0.0442 (3)	0.6921 (8)	0.0932 (3)	0.0616 (13)	
H2	0.0358	0.8249	0.0654	0.074*	
C3	−0.0053 (3)	0.6511 (10)	0.1376 (3)	0.0723 (16)	
H3	−0.0474	0.7553	0.1396	0.087*	
C4	0.0075 (4)	0.4553 (11)	0.1792 (3)	0.0750 (16)	
H4	−0.0267	0.4262	0.2087	0.090*	
C5	0.0711 (3)	0.3018 (9)	0.1771 (3)	0.0586 (13)	
H5	0.0804	0.1709	0.2061	0.070*	
C6	0.1212 (3)	0.3424 (7)	0.1318 (2)	0.0430 (10)	
C7	0.1904 (3)	0.1718 (7)	0.1323 (2)	0.0451 (10)	
C8	0.2038 (3)	0.1350 (7)	0.0618 (2)	0.0435 (10)	
H8	0.1709	0.2233	0.0177	0.052*	
C9	0.2619 (3)	−0.0218 (7)	0.0543 (2)	0.0421 (10)	
C10	0.2628 (3)	−0.0622 (7)	−0.0250 (2)	0.0418 (10)	
C11	0.2382 (3)	−0.2677 (8)	−0.0616 (2)	0.0521 (12)	
H11	0.2253	−0.3840	−0.0346	0.063*	
C12	0.2325 (3)	−0.3011 (8)	−0.1375 (2)	0.0585 (13)	
H12	0.2151	−0.4388	−0.1619	0.070*	
C13	0.2526 (3)	−0.1323 (9)	−0.1769 (3)	0.0611 (13)	
H13	0.2491	−0.1554	−0.2281	0.073*	
C14	0.2776 (4)	0.0702 (9)	−0.1418 (3)	0.0690 (15)	
H14	0.2913	0.1846	−0.1690	0.083*	
C15	0.2828 (3)	0.1064 (8)	−0.0654 (3)	0.0587 (13)	
H15	0.2997	0.2450	−0.0418	0.070*	
C16	0.3797 (3)	−0.3154 (7)	0.1242 (2)	0.0458 (11)	
C17	0.3834 (3)	−0.5000 (7)	0.1687 (2)	0.0536 (12)	
H17	0.3408	−0.5166	0.1910	0.064*	
C18	0.4486 (4)	−0.6602 (8)	0.1808 (3)	0.0679 (15)	
H18	0.4510	−0.7834	0.2120	0.082*	
C19	0.5104 (4)	−0.6391 (10)	0.1473 (3)	0.0804 (17)	
H19	0.5539	−0.7497	0.1544	0.096*	
C20	0.5083 (3)	−0.4551 (12)	0.1031 (3)	0.0783 (17)	
H20	0.5509	−0.4409	0.0807	0.094*	
C21	0.4432 (3)	−0.2894 (9)	0.0914 (3)	0.0647 (13)	
H21	0.4424	−0.1633	0.0620	0.078*	

### Atomic displacement parameters (Å^2^)

**Table:**

	*U*^11^	*U*^22^	*U*^33^	*U*^12^	*U*^13^	*U*^23^
O1	0.077 (2)	0.063 (2)	0.0374 (15)	0.0107 (17)	0.0279 (15)	0.0051 (15)
N1	0.062 (2)	0.054 (2)	0.0368 (17)	0.0121 (18)	0.0251 (17)	0.0093 (16)
C1	0.056 (3)	0.053 (3)	0.047 (2)	−0.005 (2)	0.023 (2)	−0.006 (2)
C2	0.067 (3)	0.053 (3)	0.056 (3)	0.005 (2)	0.016 (2)	−0.007 (2)
C3	0.058 (3)	0.091 (4)	0.064 (3)	0.012 (3)	0.021 (3)	−0.023 (3)
C4	0.069 (3)	0.104 (5)	0.065 (3)	0.013 (3)	0.041 (3)	−0.002 (3)
C5	0.068 (3)	0.066 (3)	0.048 (2)	0.004 (3)	0.031 (2)	0.000 (2)
C6	0.051 (2)	0.046 (2)	0.0301 (19)	−0.0043 (19)	0.0157 (18)	−0.0060 (17)
C7	0.051 (2)	0.047 (3)	0.040 (2)	−0.005 (2)	0.0211 (19)	−0.0032 (19)
C8	0.054 (2)	0.047 (2)	0.0299 (19)	0.002 (2)	0.0177 (18)	0.0031 (18)
C9	0.050 (2)	0.044 (2)	0.0333 (19)	−0.001 (2)	0.0178 (18)	0.0006 (18)
C10	0.047 (2)	0.045 (2)	0.0315 (19)	0.0042 (19)	0.0152 (18)	0.0012 (17)
C11	0.070 (3)	0.050 (3)	0.041 (2)	−0.006 (2)	0.028 (2)	−0.004 (2)
C12	0.071 (3)	0.061 (3)	0.044 (2)	−0.004 (2)	0.024 (2)	−0.015 (2)
C13	0.078 (3)	0.073 (4)	0.038 (2)	0.012 (3)	0.029 (2)	0.003 (2)
C14	0.104 (4)	0.067 (4)	0.051 (3)	0.009 (3)	0.046 (3)	0.017 (3)
C15	0.094 (4)	0.046 (3)	0.047 (2)	0.000 (2)	0.040 (3)	0.004 (2)
C16	0.048 (2)	0.051 (3)	0.033 (2)	0.002 (2)	0.0113 (18)	−0.0049 (18)
C17	0.055 (3)	0.049 (3)	0.045 (2)	−0.004 (2)	0.008 (2)	−0.003 (2)
C18	0.068 (3)	0.042 (3)	0.068 (3)	0.004 (3)	0.002 (3)	0.002 (2)
C19	0.068 (4)	0.076 (4)	0.078 (4)	0.024 (3)	0.011 (3)	−0.012 (3)
C20	0.057 (3)	0.114 (5)	0.064 (3)	0.016 (3)	0.025 (3)	−0.003 (3)
C21	0.056 (3)	0.084 (4)	0.056 (3)	0.007 (3)	0.024 (2)	0.006 (3)

### Geometric parameters (Å, °)

**Table:**

O1—C7	1.252 (5)	C10—C11	1.388 (6)
N1—C9	1.347 (5)	C11—C12	1.377 (6)
N1—C16	1.416 (5)	C11—H11	0.9300
N1—H1	0.8600	C12—C13	1.362 (6)
C1—C6	1.373 (6)	C12—H12	0.9300
C1—C2	1.386 (6)	C13—C14	1.362 (7)
C1—H1A	0.9300	C13—H13	0.9300
C2—C3	1.369 (7)	C14—C15	1.391 (6)
C2—H2	0.9300	C14—H14	0.9300
C3—C4	1.377 (8)	C15—H15	0.9300
C3—H3	0.9300	C16—C17	1.369 (6)
C4—C5	1.382 (7)	C16—C21	1.384 (6)
C4—H4	0.9300	C17—C18	1.366 (7)
C5—C6	1.393 (6)	C17—H17	0.9300
C5—H5	0.9300	C18—C19	1.363 (8)
C6—C7	1.502 (6)	C18—H18	0.9300
C7—C8	1.416 (5)	C19—C20	1.369 (8)
C8—C9	1.368 (6)	C19—H19	0.9300
C8—H8	0.9300	C20—C21	1.389 (7)
C9—C10	1.486 (5)	C20—H20	0.9300
C10—C15	1.372 (6)	C21—H21	0.9300
			
C9—N1—C16	130.5 (3)	C12—C11—C10	120.6 (4)
C9—N1—H1	114.7	C12—C11—H11	119.7
C16—N1—H1	114.7	C10—C11—H11	119.7
C6—C1—C2	120.4 (4)	C13—C12—C11	119.9 (4)
C6—C1—H1A	119.8	C13—C12—H12	120.0
C2—C1—H1A	119.8	C11—C12—H12	120.0
C3—C2—C1	120.4 (5)	C12—C13—C14	120.3 (4)
C3—C2—H2	119.8	C12—C13—H13	119.8
C1—C2—H2	119.8	C14—C13—H13	119.8
C2—C3—C4	119.8 (5)	C13—C14—C15	120.2 (4)
C2—C3—H3	120.1	C13—C14—H14	119.9
C4—C3—H3	120.1	C15—C14—H14	119.9
C3—C4—C5	120.1 (5)	C10—C15—C14	120.1 (4)
C3—C4—H4	120.0	C10—C15—H15	120.0
C5—C4—H4	120.0	C14—C15—H15	120.0
C4—C5—C6	120.3 (5)	C17—C16—C21	119.7 (4)
C4—C5—H5	119.9	C17—C16—N1	118.0 (4)
C6—C5—H5	119.9	C21—C16—N1	122.3 (4)
C1—C6—C5	119.0 (4)	C18—C17—C16	121.1 (5)
C1—C6—C7	122.8 (4)	C18—C17—H17	119.5
C5—C6—C7	118.3 (4)	C16—C17—H17	119.5
O1—C7—C8	123.2 (4)	C19—C18—C17	119.9 (5)
O1—C7—C6	117.5 (3)	C19—C18—H18	120.0
C8—C7—C6	119.3 (4)	C17—C18—H18	120.0
C9—C8—C7	124.4 (4)	C18—C19—C20	120.0 (5)
C9—C8—H8	117.8	C18—C19—H19	120.0
C7—C8—H8	117.8	C20—C19—H19	120.0
N1—C9—C8	120.4 (3)	C19—C20—C21	120.6 (5)
N1—C9—C10	119.7 (4)	C19—C20—H20	119.7
C8—C9—C10	119.6 (3)	C21—C20—H20	119.7
C15—C10—C11	118.8 (4)	C16—C21—C20	118.7 (5)
C15—C10—C9	120.6 (4)	C16—C21—H21	120.6
C11—C10—C9	120.5 (4)	C20—C21—H21	120.6
			
C6—C1—C2—C3	−1.5 (7)	N1—C9—C10—C11	56.6 (6)
C1—C2—C3—C4	0.4 (7)	C8—C9—C10—C11	−117.8 (5)
C2—C3—C4—C5	0.9 (8)	C15—C10—C11—C12	−0.9 (7)
C3—C4—C5—C6	−1.2 (7)	C9—C10—C11—C12	175.3 (4)
C2—C1—C6—C5	1.2 (6)	C10—C11—C12—C13	1.0 (7)
C2—C1—C6—C7	−177.8 (4)	C11—C12—C13—C14	−0.4 (8)
C4—C5—C6—C1	0.2 (6)	C12—C13—C14—C15	−0.1 (8)
C4—C5—C6—C7	179.2 (4)	C11—C10—C15—C14	0.3 (7)
C1—C6—C7—O1	146.2 (4)	C9—C10—C15—C14	−175.9 (4)
C5—C6—C7—O1	−32.8 (6)	C13—C14—C15—C10	0.2 (8)
C1—C6—C7—C8	−35.9 (6)	C9—N1—C16—C17	−143.8 (4)
C5—C6—C7—C8	145.0 (4)	C9—N1—C16—C21	38.9 (7)
O1—C7—C8—C9	0.5 (7)	C21—C16—C17—C18	−0.4 (6)
C6—C7—C8—C9	−177.2 (4)	N1—C16—C17—C18	−177.7 (4)
C16—N1—C9—C8	−176.4 (4)	C16—C17—C18—C19	−1.1 (7)
C16—N1—C9—C10	9.3 (7)	C17—C18—C19—C20	1.6 (8)
C7—C8—C9—N1	−1.0 (6)	C18—C19—C20—C21	−0.5 (8)
C7—C8—C9—C10	173.4 (4)	C17—C16—C21—C20	1.4 (7)
N1—C9—C10—C15	−127.2 (5)	N1—C16—C21—C20	178.6 (4)
C8—C9—C10—C15	58.4 (6)	C19—C20—C21—C16	−0.9 (8)

### Hydrogen-bond geometry (Å, °)

**Table:**

*D*—H···*A*	*D*—H	H···*A*	*D*···*A*	*D*—H···*A*
N1—H1···O1	0.86	1.94	2.643 (5)	138
C18—H18···Cg^i^	0.93	2.84	3.627 (1)	142

Symmetry codes: (i) −*x*+1, *y*−1/2, −*z*+1/2.
